# EV‐Checklist: AI‐Powered Rapid Documentation for Enhancing Transparency and Accessibility of Extracellular Vesicle Research Data

**DOI:** 10.1002/jev2.70343

**Published:** 2026-07-13

**Authors:** Rodolphe Poupardin, Martin Wolf, Antri Stefani, Gregor Fuhrmann, Dirk Strunk

**Affiliations:** ^1^ Cell Therapy Institute Paracelsus Medical Private University Salzburg Austria; ^2^ Austrian Red Cross Research OeRK Vienna Austria; ^3^ Department of Biology Friedrich‐Alexander‐University Erlangen‐Nürnberg Erlangen Germany

**Keywords:** artificial intelligence (AI), checklist, dynamic reporting, extracellular vesicles (EVs), MISEV, research transparency

## Abstract

Transition of extracellular vesicle (EV) research from basic discovery to clinical application raised hopes regarding diagnostic, therapeutic and prognostic progress. Rigorous reporting of experimental details is required to align the EV field with pharmaceutical quality standards. MISEV2023 recommendations encourage concise reporting but widespread adoption remains limited. Current reporting tools are time‐consuming, and adherence declines despite rapidly growing number of EV studies. We therefore created EV‐Checklist, a complementary digital tool that streamlines reporting and increases transparency. By uploading manuscript text, an AI‐assisted algorithm automatically completes a checklist covering EV nomenclature, source, isolation, characterization and function. To ensure accuracy, users validate AI‐generated entries before submission—ideally, gaps in reporting can be closed (e.g., missing particle/protein ratio). The resulting concise report can accompany manuscripts helping editors, reviewers and readers by presenting key methodological and results details at a glance. EV‐Checklist complements existing comprehensive registries as ‘fast‐and‐easy’ tool enhancing clarity and accessibility of EV research data and may promote higher adherence to documentation standards. Adoption may be encouraged by journal endorsement to streamline the review process for compliant submissions, signalling adherence to MISEV2023 standards. EV‐Checklist and an accompanying AI‐assisted search tool (PMC EV Search), spanning over 45,700 open‐access EV manuscripts, are publicly available at https://ev‐zone.org/.

## Graphical abstract

The EV‐Checklist tool facilitates comprehensive AI‐assisted standardized reporting, aligned with MISEV2023 recommendations, by extracting information on nomenclature, source, separation/concentration, characterization and properly controlled functional studies of EVs, instantly after manuscript upload at https://ev‐zone.org.

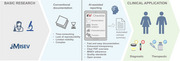

## 1 Introduction

**FIGURE 1:**
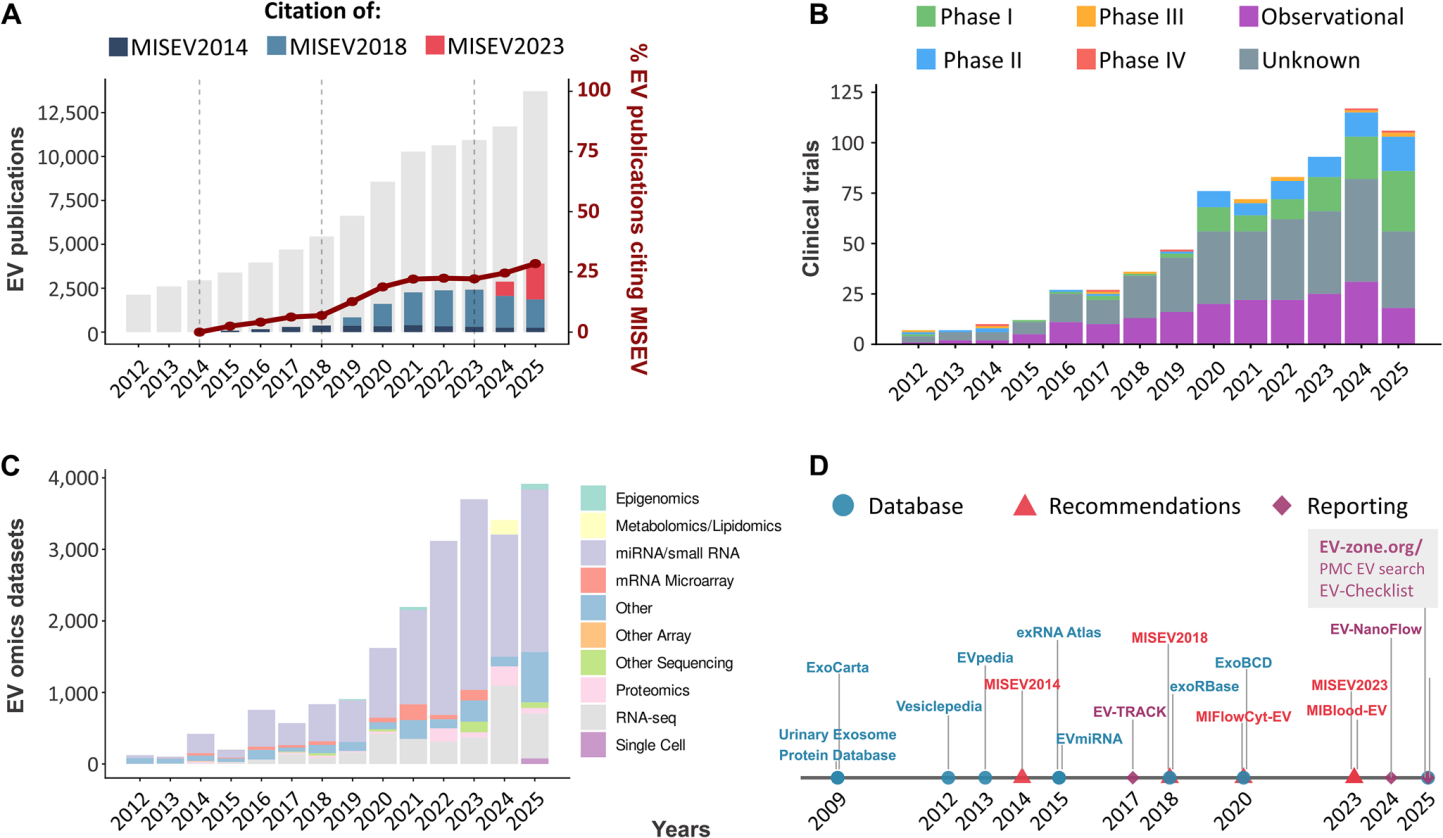
**Overview of the EV field for 2012–2025**. (**A**) Cumulative proportion of publications citing MISEV relative to all articles related to EVs published per year. (**B**) Clinical trials registered at Clinicaltrials.org using EVs for diagnostic or therapeutic approaches. **(C)** Accumulation of omics datasets for EVs acquired from the GEO‐NCBI EV database; different technologies depicted according to colour code. (**D**) Chronological development of EV reporting tools and platforms to be compiled in the EV‐Checklist.

EVs have emerged as popular research topic due to their role in intercellular communication and their potential as theranostic biomarkers and therapeutic agents including their use as drug delivery vehicles (Berumen Sánchez et al., 2021; Kalluri and LeBleu, 2020; Nguyen et al., 2020; Van Niel et al., 2018; Yáñez‐Mó et al., 2015). The EV research field grew from 2,126 publications in 2012 to 13,715 in 2025 (Poupardin et al., 2024a, Poupardin et al., 2021) (Figure 1A). Meanwhile clinical trials registered at https://clinicaltrials.gov for testing the use of EVs as therapeutic agent or for diagnostic purpose increased from seven in 2012 to 117 in 2024 (data retrieved 10.04.2026) (Mizenko et al., 2024). The rise in phase I and II trials since 2022 reflects the ongoing transition of EV research from basic discovery toward clinical application (Figure 1B). Parallel to this, the complexity of EV research has intensified, evidenced by the exponential increase in multi‐omics datasets—including RNA‐seq and proteomics—deposited in public databases (Figure 1C). These developments introduced significant challenges, including stringent quality control requirements and adherence to pharmaceutical documentation standards. While good laboratory practice principles can guide academic research, the rapidly expanding literature underscores the need for accessible, concise, and standardized reporting of key experimental parameters to ensure reproducibility and transparency. Establishing recommendations for ‘Minimal Information for Studies of Extracellular Vesicles’ (MISEV) in 2013 marked a milestone, advocating for rigorous reporting to enhance transparency and comparability across studies in the EV field (Lötvall et al., 2014). These recommendations have been updated in a growing community approach in 2018 and 2023 (Théry et al., 2018; Welsh et al., 2024). Citation of MISEV recommendations has grown to 26% of EV‐related publications, reflecting increasing awareness. The actual level of adherence to recommended reporting standards remains difficult to assess.

Recognizing this lack of adherence to recommended reporting, we developed an EV‐Checklist to facilitate a swift documentation process covering important aspects of EV research highlighted in the MISEV recommendations. We applied a preliminary version of this checklist in several manuscripts during the developmental process (Poupardin et al., 2024b; Andrade et al., 2021; Binder et al., 2021; Poupardin et al., 2021; Gomes et al., 2022; Wolf et al., 2022; Blöchl et al., 2025). Based on this experience, we created an online tool https://ev‐zone.org to make the checklist publicly available. Upon completion, this tool generates a concise PDF or HTML‐formatted checklist report providing users with a quick overview of completed and pending key characterizations. This EV‐Checklist can then be attached to manuscripts upon submission, providing clear reporting of methods and quality controls already to editors and reviewers.

## 2 Methods and Results

### 2.1 EV‐Checklist for Users: Simplicity and Ease of Use

**FIGURE 2:**
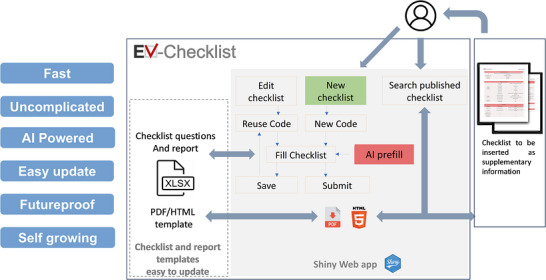
**Architecture of the EV‐Checklist online tool**. Users can initiate a new checklist by obtaining a new code or continue a saved checklist using a previously acquired code. The tool features AI‐assisted extraction that automatically pre‐fills the checklist from an uploaded text manuscript or PMCID which users then must review and validate. Upon submission, users receive formatted PDF and HTML reports that can be attached to their manuscript. All published checklists are made accessible in the checklist database. The questionnaire structure and report template are both housed within an Excel file, facilitating quick and effortless updates to the tool.

Based on MISEV2023 (Welsh et al., 2024) we divided the checklist into five categories: General information (including application and nomenclature, sample information (source, sample preparation and pre‐analytics), isolation methods, characterization (including cargo) and function. We further included two optional sections: other ‘information’, where users can provide supplementary details relevant to the study, and a ‘feedback’ section, only visible to the website administrator, where users can offer suggestions for improving EV‐Checklist. When initially accessing the tool, users are prompted to either generate a new unique code or enter a previously generated code. This code acts as an identifier for their checklist, enabling the save‐and‐resume functionality (Figure 2).

Based on current testing experience, manually completing the EV‐Checklist required 15 to 25 min if all information was readily available. To further reduce this time and improve adoption, we implemented an AI‐assisted extraction system that leverages recent advances in large language models (LLM). Users can upload their manuscript text file or provide a PMCID if the manuscript is published already and is freely accessible on PubMed. The AI algorithm then automatically parses the entire manuscript including text embedded within tables in the main manuscript body to identify key information including general aspects such as EV nomenclature, source material, isolation methods, characterization techniques, and functional assays, and to pre‐fill the corresponding checklist entries. Importantly, the AI extraction utilizes a secure server‐side API call. Uploaded manuscripts are processed temporarily for extraction and are not stored or used to train the underlying AI models, ensuring the confidentiality and intellectual property of unpublished research.

**FIGURE 3:**
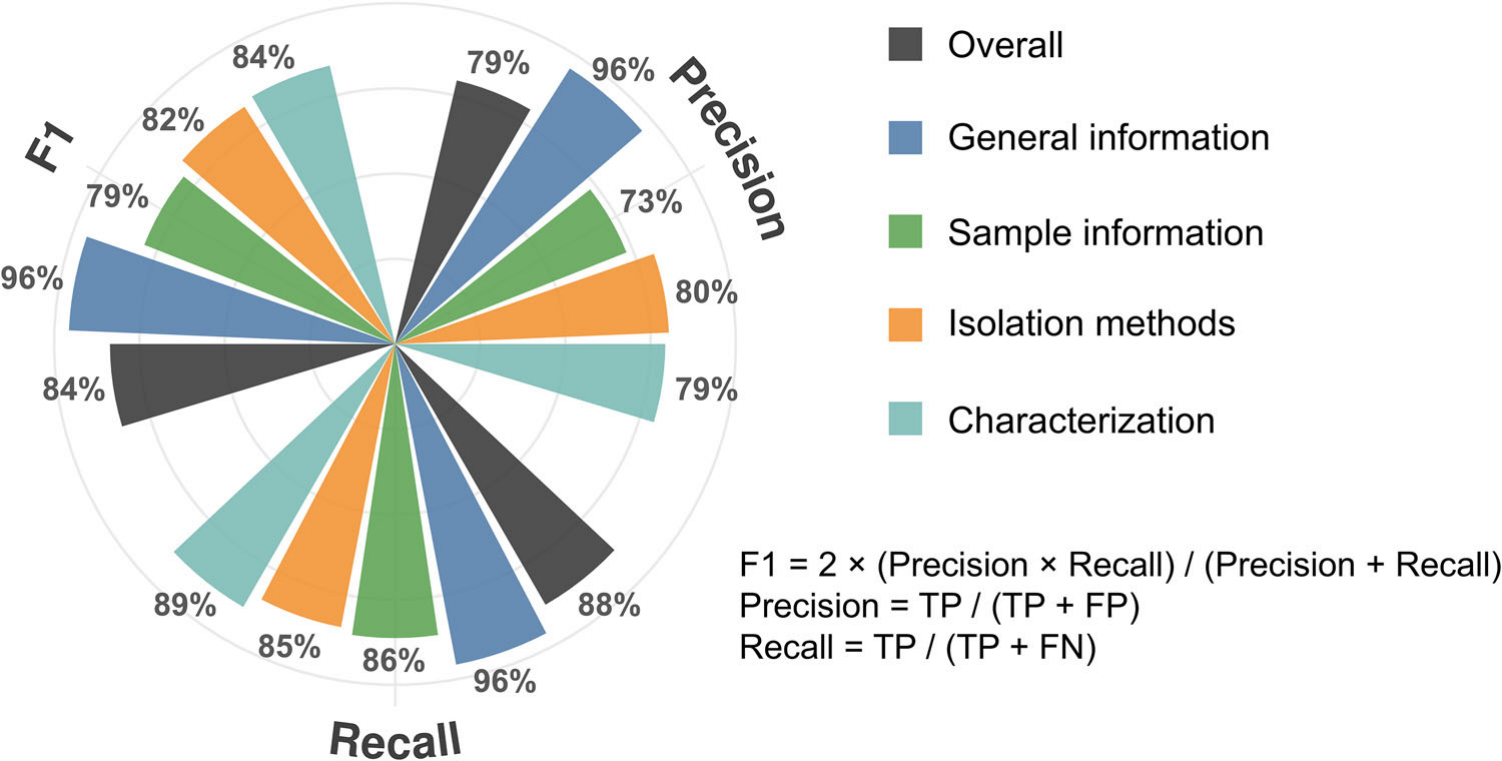
**Validation of AI‐assisted data extraction against the EV‐TRACK database**. Radar chart visualizing the precision (percentage of AI‐retrieved fields that correctly matched the ground truth, reflecting a minimized false‐positive rate), recall (percentage of actual fields successfully found by the AI, reflecting a minimized false‐negative rate), and F1 Score (harmonic mean of precision and recall) of the EV‐Checklist AI algorithm. Performance is categorized across four core methodological sections (general info, sample info, isolation, and characterization) alongside the overall average. AI‐extracted parameters were systematically evaluated against manually curated, ground truth records from the EV‐TRACK database.

After the AI algorithm completed the initial extraction, users are requested to review and validate or modify the pre‐filled responses before generating the final report. This approach combines the efficiency of automated extraction with the reliability of expert human oversight, substantially reducing manual data entry time while maintaining scientific rigor. As quantitative evaluation of LLM performance is currently a heavily debated issue in data science we decided to use a simple approach to evaluate the correctness of AI‐filled checklists tailored to this specific task (Rasool et al., 2024). To evaluate the accuracy and robustness of the EV‐Checklist AI extraction algorithm at a broader scale, we conducted a systematic validation against manually curated data from the EV‐TRACK database. A testing dataset comprising 35 open‐access EV publications with corresponding EV‐TRACK records was assembled. Full text of these articles was automatically retrieved via the PubMed Central (PMC) Open Access API and processed through our automated LLM pipeline (Gemini 3.0 Flash, temperature = 0.05). The temperature parameter, which controls the randomness of model outputs, was chosen near zero to ensure highly deterministic and conservative responses, reducing the likelihood of the AI generating information not present in the manuscript. For each manuscript, 20 standardized fields were compared between AI‐extracted values and EV‐TRACK entries using a semantic equivalence judge (fuzzy text matching, numeric extraction, and secondary LLM semantic review). Fields were grouped into four categories: General information, sample information, isolation methods, and characterization. Performance was evaluated using precision, recall, and F1 score (Figure 3). Overall, the system achieved a precision of 79%, recall of 88%, and F1 score of 84%. Performance was highest for general information fields (precision 96%, recall 96%, F1 96%), which included well‐defined variables such as EV nomenclature and study application. Isolation methods and characterization fields showed balanced intermediate performance (F1: 82% and 84%, respectively). Sample information fields yielded modest precision (73%), likely reflecting greater variability in how cell type, harvesting conditions, and culture medium are reported across publications. The consistently high recall across all categories (85–96%) indicates that the system reliably extracts a value present in the ground truth. Precision losses primarily arose from differences in reporting granularity or terminology (Figure 3, Table S1). AI‐driven results will be further optimized during platform operation thus reducing manual input time to 5–10 min for the user.

**FIGURE 4:**
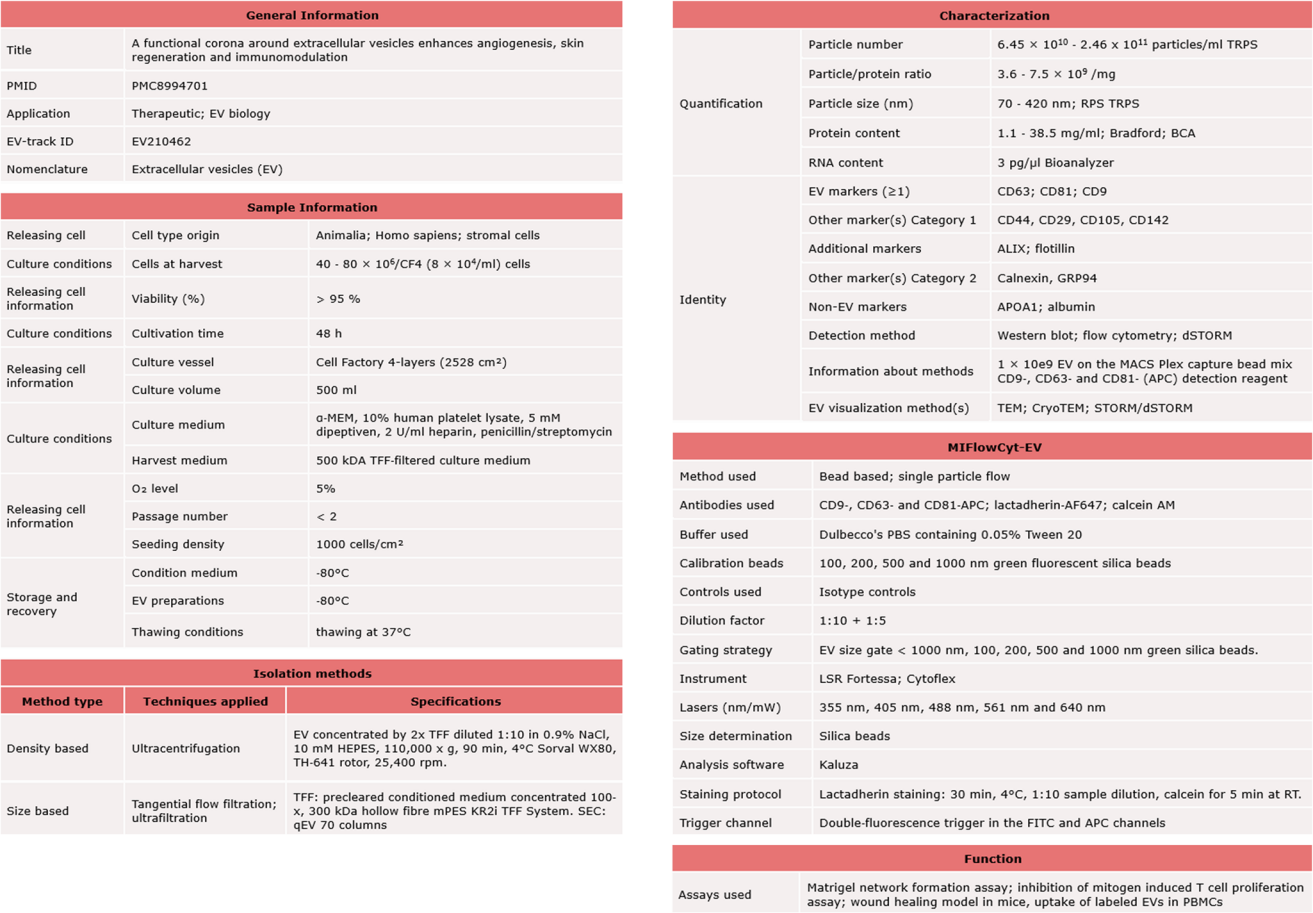
**Example of EV‐Checklist PDF output**. The checklist is divided into five mandatory categories: General information (including nomenclature), sample information (including collection and storage), isolation methods, characterization, and function. MIFlowCyte‐EV has been included here optionally. Additional optional categories like MIBlood‐EV and other information are available in addition.

To facilitate intuitive navigation, we organized the checklist into several tabs, boxes and logically grouped related topics (Figure S1). Questions within the checklist are dynamically displayed based on the user's responses to previous questions. The EV‐Checklist output is provided as a PDF and HTML format summary table ensuring a high‐quality printable output that meets standards for publication (Figure 4).

We host the EV‐Checklist on the platform https://ev‐zone.org that also provides other useful EV tools like a database of all EV‐related publications from PubMed Central with an advanced Regex and AI search tool. The EV‐zone platform is hosted by a commercial cloud provider in Germany. To ensure scalability and manage concurrent AI extraction requests effectively, the tools operate within multiple docker containers orchestrated behind a NGINX load balancer. The PMC EV literature database is updated weekly via automated retrieval from PubMed Central, allowing users to search across >45,700 entries. An integral feature of EV‐Checklist is the checklist database, which serves as a repository of all submitted checklists (> 50) marked as published. Checklists from unpublished manuscripts remain strictly private and are accessible only for the authors via their unique checklist code. Once a manuscript is accepted, authors can easily update their checklist status to ‘published’ by providing a DOI or PMID. This published repository presents a structured view of the diverse studies conducted in the EV field, promoting visibility of published works, and facilitating a collaborative and open research environment.

### 2.2 EV‐Checklist for Developers: A Tool Optimized to be Future Proof

Utilizing the Shiny framework in the programming language R, known for creating user‐friendly interfaces while maintaining a robust backend, we developed a highly interactive web application. The checklist structure and report template are both stored in a structured Excel file, which serves as a dynamic configuration system for both the questionnaire and the generated output. This design choice offers several advantages: First, the Excel file allows for easy modifications and additions to the checklist by the administrator without the necessity for code alterations. New questions can be added, or existing ones can be modified by updating the excel file. Second, the Excel file defines the types of input fields associated with each question, supporting various types of questions including text fields, radio buttons, and checkboxes. This flexibility accommodates a variety of data inputs, aiding in capturing precise and structured responses from the users. Furthermore, questions are flagged as either required or optional within the Excel file, ensuring that critical information is not omitted and guiding users to provide all required details before submission. The Excel file also supports the definition of dependencies between questions. Some questions may only be relevant based on the responses to previous questions. Questions are grouped into boxes and tabs based on thematic or procedural similarities, facilitating intuitive navigation through the checklist and making it easier for users to complete and review their entries. The Excel file also defines display parameters for the report, including section headers, column layouts, and custom labels for clearer presentation in the final output. This Excel‐driven approach promotes a high degree of customization and adaptability, ensuring that the tool remains relevant and useful as the standards and requirements for EV research documentation evolve over time without requiring changes to the codebase.

Administrators can update questionnaire content, modify report layouts, and adjust formatting specifications entirely through the Excel file, making the tool highly maintainable and future‐proof.

A key future development will leverage the AI extraction capabilities to systematically mine the open‐access EV literature and automatically increase the database. We plan to implement an automated pipeline that processes open‐access publications from PubMed Central (PMC), using the same large language model‐based extraction algorithms currently employed for individual manuscript uploads. This system will automatically extract methodological details and generate standardized checklist entries for each study. To maintain transparency and data quality, all database entries will be clearly labelled by their source, that is, user‐submitted entries (manually reviewed and validated by the authors) versus AI‐extracted entries (automatically generated from published literature). Such a resource will be vital for systematic reviews, enabling researchers to rapidly identify studies using specific methodologies or meeting quality criteria. Furthermore, it will support meta‐analyses by offering standardized data on isolation methods, characterization techniques, and experimental conditions across hundreds or thousands of studies (Poupardin et al.,2024a).

## 3 Discussion

The MISEV recommendations encouraged authors to document key parameters of their EV studies, including nomenclature, collection and pre‐processing of source material, storage conditions, isolation and concentration methods, characterization of EV preparations, information on EV release and uptake, and detailed descriptions of functional assays or in vivo studies where applicable. Over the past years, researchers increasingly reported three or four EV characterization categories, mostly reporting markers of plasma membrane and/or endosomal proteins of EV identity such as CD63, CD81 and CD9 (category I) and cytosolic proteins including actin, glyceraldehyde‐ 3‐phosphate dehydrogenase (GAPDH) and tumour susceptibility gene 101 (TSG101; category II). At least two markers out of two different categories were used to identify EVs in 48.5% of publications indicating growing EV characterization efforts (Poupardin et al., 2021). However, in a recent analysis of research on ‘EV and heparin’, we found that 82.2% of publications in this area (150/180) did not provide any EV quantity data. Of the minority that did report quantification, most reported particles/volume (29/180 studies, 16.1%), while only 1.7% (3/180) reported particle/protein ratio as recommended by MISEV2023, highlighting a reporting gap in the literature and a lack of scientific rigor bringing a negative view on the field and its findings (Andrade et al., manuscript under revision). In line with these developments, new task force initiatives have been launched to promote method‐specific reporting standards. MIFlowCyt‐EV (Welsh et al., 2020) and the NanoFlow repository (Arce et al., 2023) offer a framework for standardized reporting of EV flow cytometry experiments. MIBlood‐EV (Lucien et al., 2023) recently focused on enhancing transparency in reporting pre‐analytical parameters for blood collection, quality control of plasma and serum parameters, processing, and storage. It was developed in response to surveys from International Society for Extracellular Vesicles (ISEV) members and the blood EV task force. These recommendations aim to improve biobank quality, facilitate sample exchange between biobanks and labs, and enhance comparative EV studies and peer review processes. Initially provided as a user‐friendly document, there is the possibility to upload the filled forms to a centralized database, restricted to the MIBlood‐EV checklist. Building upon these efforts, the ISEV has extended its guidance to cerebrospinal fluid (CSF)‐EV studies (Sandau et al., 2024). This publication introduced comprehensive recommendations for the reproducibility of CSF‐EV studies and provides a detailed checklist template. The template is designed for researchers to report key methods and some results, which can be inserted alongside the manuscript during submission, ensuring a standardized approach to documenting CSF‐EV research practices. There is no database provided for researchers to upload their CSF checklists. In addition to these targeted initiatives, efforts have been made using surveys (Royo et al., 2020) and large‐scale text mining approaches to systematically analyze and understand the methodologies employed in the field (Poupardin et al., 2024a, 2021). They identified gaps in method reporting and initiated the development of open access MISEV‐adherent online tools that ease reporting.

These initiatives encourage more detailed reporting, contributing to rigor and reproducibility in the field. EV‐TRACK promotes comprehensive documentation through its EV‐metric scoring system, providing the most complete reporting platform currently available (Van Deun et al., 2017). The advantage of detailed data submission to EV‐TRACK by reporting each EV preparation separately requires a time‐intense submission process. The team behind EV‐TRACK is currently working to streamline the data submission process aiming for a more user‐friendly version. Similarly, ExoCarta (http://exocarta.org/), Vesiclepedia (http://microvesicles.org/) and other EV databases offer valuable resources for sharing and comparing EV data (Keerthikumar et al., 2016; Chitti et al., 2024) (Figure 1D).

The modular design of EV‐Checklist can host other questionnaires pertinent to the research community. In addition to its current capabilities, the tool can be aligned with specialized checklists from various task force initiatives. For instance, when a user selects ‘blood’ as a sample source, the tool can prompt additional questions relevant to blood EV research, in accordance with MIBlood‐EV guidelines (Lucien et al., 2023). We envision EV‐zone as an entry hub that connects users toward more detailed, specialized reporting platforms. The platform features an ‘Enhance your EV reporting’ panel that dynamically generates upon checklist submission. This section provides direct links to specialized frameworks such as MIFlowCyt‐EV and MIBlood‐EV. All users are encouraged to submit comprehensive data to EV‐TRACK. Thereby, it can easily be adapted to future advancements and to support a broader spectrum of informational and data collection needs.

Implementing such an EV‐Checklist tool within the manuscript submission process could provide a concise overview of methods and results for editors and reviewers thus supporting decision processes. Initiatives such as Cell Press’ STAR Methods or Nature's Reporting Summary have promoted structured, transparent, and accessible reporting in life sciences. Both provide a structured format for authors to detail their experimental procedures, aiming to enhance transparency, reproducibility, and accessibility of research findings (Tonzani and Fiorani, 2021).

Taken together, a streamlined time‐efficient documentation tool that covers EV research data of a given study at once would be beneficial. EV‐Checklist has been designed to complement these established registries as an AI‐powered rapid but comprehensive reporting tool that may serve as an entry point for researchers and may support editors and reviewers, potentially also encouraging subsequent submission of detailed data to specialized platforms.

## 4 Conclusion

We developed a comprehensive open access tool combining AI‐assisted completion with user validation to balance efficiency and accuracy, while generating automated summaries to streamline documentation of EV research. Additionally, the publicly accessible checklist database further augments the transparency and visibility of published data. Its scalable design can accommodate additional community questionnaires, positioning it as an adaptable resource for standardized reporting. However, without measurable incentive for authors for providing EV‐TRACK or EV‐Checklist or any other concise reporting, improvements towards data transparency and accessibility in EV research might be limited. Concise reporting needs to be requested by editors or publishers during the submission process, to streamline the decision process and to support quality check by their voluntary reviewers.

## Author Contributions

**Rodolphe Poupardin**: conceptualization, methodology, software, investigation, visualization, writing – review and editing, writing – original draft, formal analysis, validation. **Martin Wolf**: conceptualization, investigation, validation, formal analysis, visualization, data curation, writing – original draft, writing – review and editing, methodology. **Antri Stefani**: visualization, writing – review and editing, formal analysis. **Gregor Fuhrmann**: writing – review and editing, writing – original draft. **Dirk Strunk**: project administration, resources, supervision, writing – original draft, writing – review and editing, funding acquisition, formal analysis, conceptualization, investigation, visualization, methodology.

## Funding

This work was supported by funding from the European Union's Horizon Europe research and innovation program (grant agreement 101095635 PROTO, 101080267 NEXGEN‐PD and 101056712 HEAL to DS), by Land Salzburg (20102‐F2001080‐FPR, 20102‐F2400472 Cancer Cluster II+III, 20102‐F2100572‐FPR “EV‐Quant”, 20102/F2400948‐FPR “Good Fibration” and P2506840 “AI‐BOOST”) to DS and from the European Research Council (ERC) under the European Union's Horizon 2020 research and innovation program (grant agreement 945602, Gels4Bac to GF)

## Conflicts of Interest

The authors have declared no financial conflict of interest.

## Supporting information




**Supplementary Table S1**: Validation of AI‐extracted EV checklist data against EV‐TRACK. Comparison between values extracted by the AI‐powered EV checklist system and the corresponding manually curated entries in EV‐TRACK, for all 35 benchmarked manuscripts. Each row represents a single field‐paper comparison. Columns are defined as follows: PubMed Central identifier (PMCID) of the manuscript; evtrack_id; field_label, standardized EV reporting field evaluated; evtrack_field and checklist_field, internal field names in EV‐TRACK and in the AI checklist, respectively; evtrack_value and checklist_value, values curated in EV‐TRACK and extracted by the AI checklist; match, initial automated string/semantic match outcome (TRUE/FALSE); score, continuous agreement score between 0 and 1 (1 = full match, 0 = no match, intermediate values = partial match, for example, overlapping items in lists of markers or methods); error_type, initial classification as TP (true positive: both systems agree on a valid value), FP (false positive: AI extracted a value where EV‐TRACK had none or “Not determined”), FN (false negative: AI missed a value present in EV‐TRACK), TN (true negative: both systems report absence), Discordant (conflicting values), or Mapping (different granularity or category); note, automated rationale generated during string‐level comparison; llm_match, final match decision after LLM‐based semantic review (judge pass), accounting for synonyms, abbreviations, equivalent terminology, and differences in reporting granularity (TRUE/FALSE); llm_reason, explanation provided by the LLM judge when the semantic review overrode the initial automated match; llm_error_type, final error classification after LLM review, used for downstream computation of Precision,Recall, and F1 (Figure 3).

**Supplementary Figure S1: EV‐Checklist online screenshots: (A)** AI‐assisted questionnaire divided into tabs: Nomenclature/application, EV sources, isolation methods, characterization, function and ‘other’. **(B)** Fully searchable database for published EV‐Checklists with possibility to export data.

## Data Availability

The data that supports the findings of this study are available in the supplementary material of this article
