# Supplementary material for: EV‐Checklist: AI‐Powered Rapid Documentation for Enhancing Transparency and Accessibility of Extracellular Vesicle Research Data
Source: J Extracell Vesicles. 2026 Jul 13;15(7):e70343. doi: 10.1002/jev2.70343 (PMC13364539; doi:10.1002/jev2.70343)
Supplement: Supplementary file 2 — Supplementary Figure S1: EV‐Checklist online screenshots: (A) AI‐assisted questionnaire divided into tabs: Nomenclature/application, EV sources, isolation methods, characterization, function and ‘other’. (B) Fully searchable database for published EV‐Checklists with possibility to export data. [file JEV2-15-e70343-s001.docx]

**Supplementary Data**

**
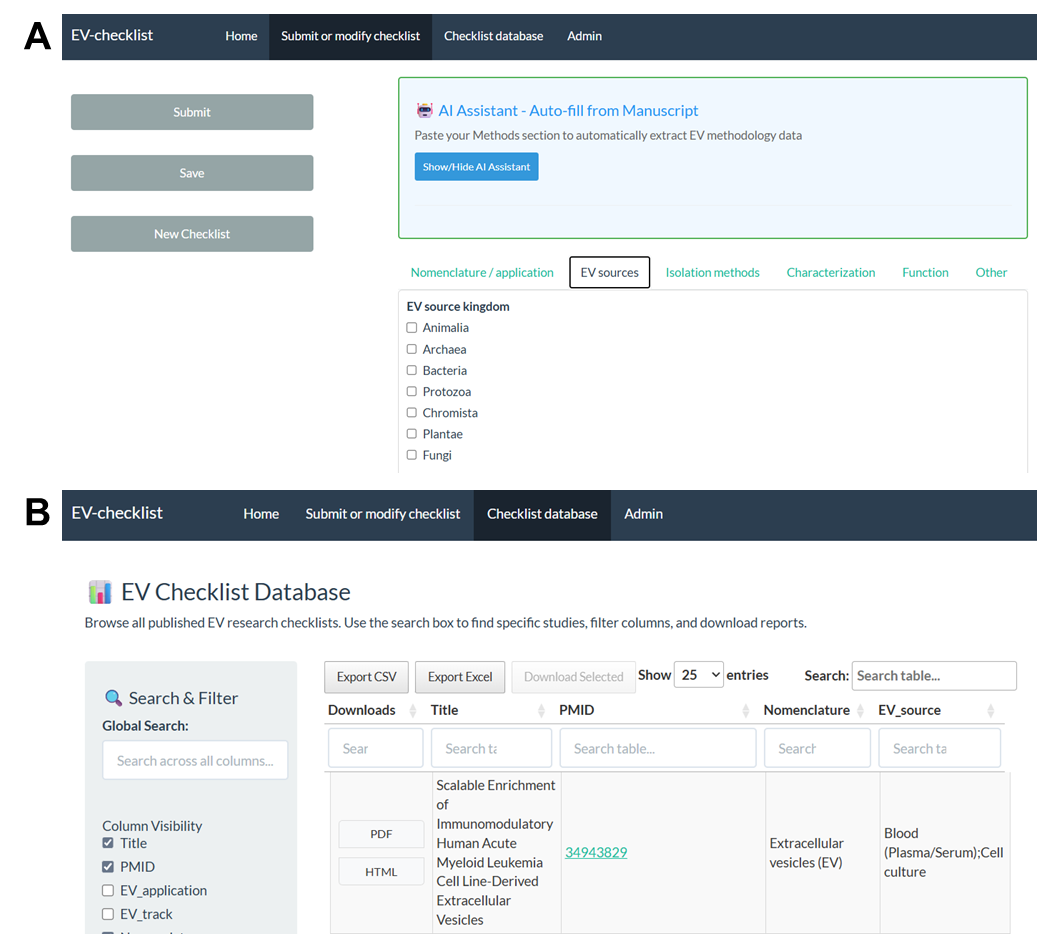
**

**Figure S1: EV-Checklist online screenshots: (A)** AI-assisted questionnaire divided into tabs: Nomenclature/application, EV sources, isolation methods, characterization, function and ‘other’. **(B)** Fully searchable database for published EV-Checklists with possibility to export data.
